# Risk of imported malaria infections in Zanzibar: a cross-sectional study

**DOI:** 10.1186/s40249-023-01129-5

**Published:** 2023-08-28

**Authors:** Bakar S. Fakih, Aurel Holzschuh, Amanda Ross, Logan Stuck, Ramadhan Abdul, Abdul-Wahid H. Al-Mafazy, Imani Irema, Abdallah Mbena, Sumaiyya G. Thawer, Shija J. Shija, Safia M. Aliy, Abdullah Ali, Günther Fink, Joshua Yukich, Manuel W. Hetzel

**Affiliations:** 1 Ifakara Health Institute, Dar es Salaam, Tanzania; 2 Swiss Tropical and Public Health Institute, Allschwil, Switzerland; 3 University of Basel, Basel, Switzerland; 4 Department of Biological Sciences, Eck Institute for Global Health, University of Notre Dame, Notre Dame, USA; 5 Tulane University School of Public Health and Tropical Medicine, New Orleans, LA USA; 6 Present Address: Amsterdam Institute for Global Health and Development, Amsterdam, Netherlands; 7 Zanzibar Malaria Elimination Programme, Zanzibar, United Republic of Tanzania

**Keywords:** Malaria, Importation, Travel, Zanzibar, Tanzania, Elimination

## Abstract

**Background:**

Zanzibar has made substantial progress in malaria control with vector control, improved diagnosis, and artemisinin-based combination therapy. Parasite prevalence in the population has remained around 1% but imported infections from mainland Tanzania contribute to sustained local transmission. Understanding travel patterns between mainland Tanzania and Zanzibar, and the risk of malaria infection, may help to control malaria importation to Zanzibar.

**Methods:**

A rolling cross-sectional survey linked to routine reactive case detection of malaria was carried out in Zanzibar between May 2017 and October 2018. Households of patients diagnosed with malaria at health facilities were surveyed and household members were tested for malaria using rapid diagnostic tests and a sub-sample by quantitative PCR (qPCR). Interviews elicited a detailed travel history of all household members who had travelled within the past two months, including trips within and outside of Zanzibar. We estimated the association of malaria infection with travel destinations in pre-defined malaria endemicity categories, trip duration, and other co-variates using logistic regression.

**Results:**

Of 17,891 survey participants, 1177 (7%) reported a recent trip, of which 769 (65%) visited mainland Tanzania. Among travellers to mainland Tanzania with travel destination details and a qPCR result available, 241/378 (64%) reported traveling to districts with a ‘high’ malaria endemicity and for 12% the highest endemicity category was ‘moderate’. Travelers to the mainland were more likely to be infected with malaria parasites (29%, 108/378) than those traveling within Zanzibar (8%, 16/206) or to other countries (6%, 2/17). Among travellers to mainland Tanzania, those visiting highly endemic districts had a higher odds of being qPCR-positive than those who travelled only to districts where malaria-endemicity was classified as low or very low (adjusted odd ratio = 7.0, 95% confidence interval: 1.9–25.5). Among travellers to the mainland, 110/378 (29%) never or only sometimes used a mosquito net during their travel.

**Conclusions:**

Strategies to reduce malaria importation to Zanzibar may benefit from identifying population groups traveling to highly endemic areas in mainland Tanzania. Targeted interventions to prevent and clear infections in these groups may be more feasible than attempting to screen and treat all travellers upon arrival in Zanzibar.

**Supplementary Information:**

The online version contains supplementary material available at 10.1186/s40249-023-01129-5.

## Background

There has been substantial progress in malaria control over the last two decades as a result of the large-scale rollout of vector control, improved diagnosis and highly effective artemisinin-based combination therapy (ACT) [1]. While there is heterogeneity in the progress made between countries, some have achieved very low transmission and are approaching malaria elimination. The E–2025 initiative, launched in April 2021, identified 25 countries with the potential to interrupt local malaria transmission by the year 2025 [2].

Movement of individuals between malaria endemic areas and receptive areas approaching elimination may result in importation of malaria parasites and contribute to sustaining local transmission, hence undermining elimination efforts [3]. Semi-immune adults who travel for work or recreation may act as asymptomatic parasite reservoirs and contribute to local transmission [4, 5]. On the other hand, non-immune travellers and migrants may have a higher risk of becoming symptomatic and suffer from more severe consequences of a malaria infection [6, 7].

Parasite importation poses a challenge to malaria elimination efforts, as previously documented in countries such as Sri Lanka [8] and China [9]. Addressing importation is therefore one of the priorities of malaria elimination programmes [10, 11]. Epidemiological information on each malaria infection that is detected in an elimination setting is required to classify the case following World Health Organization definitions as locally acquired (indigenous or introduced) or imported, and establish the extent of local transmission and importation [3]. Information on the source of imported malaria infections and human mobility data is important for designing effective intervention strategies against parasite importation [12, 13].

In Zanzibar, a semi-autonomous archipelago in the United Republic of Tanzania, renewed malaria control efforts aiming at elimination started in 2002 [14], and substantial reductions in the malaria burden have since been documented [15]. Zanzibar has achieved these reductions by scaling-up the use of long lasting insecticidal nets, indoor residual spraying, effective diagnosis using rapid diagnostic tests (RDTs) and treatment with ACT [15, 16]. As a result of these efforts, supported by substantial funding for malaria elimination, the prevalence of malaria declined to around 1% over the past decade [17–20]. Between 2010 and 2020, the annual incidence of reported clinical malaria cases has ranged between 2 and 9 per 1000 inhabitants [16, 18, 21].

An individual case-based surveillance-response system implemented since 2012 allows the Zanzibar Malaria Elimination Programme (ZAMEP) to detect every patient diagnosed with malaria in public and private health facilities in near real-time, reactively investigate whether additional cases are present in their households, and distinguish imported from locally acquired infections based on a person’s self-reported travel history [22]. A recent survey conducted in Zanzibar and using qPCR to diagnose malaria infections found that secondary infections were most likely to be found in households of clinical ‘index cases’ with a recent travel history outside of Zanzibar among co-travelling household members [23]. Malaria parasite importation by travellers between Zanzibar and mainland Tanzania has previously been described to contribute to sustaining malaria transmission in Zanzibar [24–26]. Focusing additional interventions on imported malaria cases is therefore central to preventing secondary cases and reducing local transmission [27].

In order to develop effective strategies to address the importation of malaria infections to Zanzibar, there is a need to understand relevant human behaviour [28], particularly travel patterns, and how these relate to the importation of parasites. A mathematical modelling study analysing mobile phone usage data found that malaria importation is primarily driven by traveling Zanzibar residents rather than infected visitors [25]. Yet, this study provides only limited evidence on the characteristics of travel to and from Zanzibar and how specific travel patterns may relate to malaria importation.

Building on previous analyses that found an increased risk of infection associated with travel to mainland Tanzania [23, 24], here, we investigated whether imported malaria infections found during individual case-based surveillance implemented in Zanzibar are associated with particular travel patterns in order to identify opportunities for targeting interventions to reduce the importation of malaria to Zanzibar.

## Methods

### Study setting

The study was conducted in five selected districts of Unguja [Magharibi (West) and Kusini (South)] and Pemba (Micheweni, Chake Chake and Mkoani), the two major islands of Zanzibar, United Republic of Tanzania. The total population of both islands was approximately 1.3 million in 2012 [29]. Unguja is more urban and more populated, and most of the movement crossing the Indian Ocean channel to mainland Tanzania takes place from this island. Being politically semi-autonomous, Zanzibar has its own Ministry of Health that oversees the activities of the Zanzibar Malaria Elimination Programme (ZAMEP).

In the reactive case detection (RACD) system routinely implemented by ZAMEP since 2012, clinical cases diagnosed at health facilities (‘index cases’) are notified through an electronic surveillance system and followed-up by district malaria surveillance officers (DMSO). Health facility staff send individual malaria case notifications to the system using their mobile phones. DMSOs assigned to the district where the index case resides receive the notification on their tablets and mobile phones. One of the DMSOs then visits the patients’ household to conduct malaria testing by RDT of all household members, and provide treatment to those who test positive [22].

Malaria incidence in Zanzibar exhibits clear seasonality following the bimodal rainfall patterns, with two rainy seasons in March to June and October to November [16, 30].

### Study design and survey procedures

We conducted a rolling cross-sectional survey linked to the RACD activities implemented by ZAMEP. We included index case households and, for the purpose of this study, an additional sample of four neighbouring households and five households along a 200 m transect leading away in a random direction from the index household. All members of the sampled households aged three months and above were eligible for inclusion in the study, as described in more detail elsewhere [23].

Data was collected between May 2017 and October 2018 by teams of trained local field research assistants. The research assistants accompanied DMSOs during routine households follow-ups, usually during the first follow-up of the day. They conducted the study-specific data and sample collection after the DMSO had completed their routine tasks.

Detailed information on recent travel was collected for all members of surveyed households. Information regarding their trip duration, locations visited, and their use of protective measures against mosquito bites (mosquito nets, repellents or coils) were recorded for each trip undertaken in the 60 days preceding the survey.

From all household members available on the day of the survey, a blood sample was collected from a finger-prick and tested for malaria using a RDT (SD BIOLINE Malaria Ag P.f/pan, Standard Diagnostics Inc., Giheung-gu, Yongin-si, Gyeonggi-do, Republic of South Korea) following the manufacturer’s instructions. Patients found positive by RDT were treated free of charge by the DMSOs according to national treatment guidelines. In addition, a dried blood spot (DBS) was collected on filter paper for the detection of *Plasmodium falciparum* DNA by quantitative polymerase chain reaction (qPCR), as described elsewhere [31]. Within the frame of this project, not all DBS could be analysed by qPCR. The prioritization of samples for qPCR was carried at the cluster level, and all individuals within the prioritized clusters underwent qPCR analysis.

### Participants, data management and analysis

Descriptive analyses included all individuals who reported to have travelled in the past 60 days. Since travel details were recorded only for the first trip undertaken in the 60 day period, additional trips undertaken by eight individuals who visited mainland Tanzania were not considered in these analyses. We summarized the reported travel destinations using frequencies and proportions.

Analyses of predictors of malaria infection were limited to those who reported having spent at least one night in mainland Tanzania, had their travel destination (district name) reported and had a valid qPCR result available. To identify the season during which travel occurred, we created a new variable using the start date of the first reported trip as a reference point for each study subject. Trips starting July to September were classified as occurring during dry reason, while those starting outside of this time were classified as occurring during the rainy season. To assess the association of qPCR positivity with specific predictors, we used logistic regression with a random effect to account for clustering by household. We selected covariates with a plausible association with malaria infection, including the travel destinations, the malaria endemicity in the travel destinations, trip duration, age, sex and information about the socio-economic status (SES) of households.

Malaria endemicity at district council level in mainland Tanzania was defined according to four endemicity categories (high, moderate, low, and very low) as presented in the national malaria strategic plan [32] and described in detail by Thawer et al. [33].

Wealth quintiles as an index of SES were constructed using principal component analysis (PCA) using a previously described method [34, 35]. The household-level variables used in the PCA were: source of drinking water, cooking and lighting energy, sanitation facilities, flooring material, refrigerator, and mobile phones owned by a household. Households were then grouped into the following quintiles according to the wealth index derived from the PCA: Lowest, 2nd, Middle, 4th, and Highest.

Data were analysed using STATA version 16 (StataCorp, College Station TX, USA) and QGIS software version 3.4.4.1 (https://www.qgis.org/en/site/) was used to produce choropleth maps.

## Results

### Study population

The rolling cross-sectional survey included 3473 households with 17,891 individuals, 1177 (7%) of whom reported recent travel including details of their travel destination. Of these, 379 (32%) had travelled only within Zanzibar, 769 (65%) to mainland Tanzania, and 29 (3%) outside of the United Republic of Tanzania (Additional file 1: Fig. S1). A qPCR result was available for 206 (54%) of those traveling within Zanzibar, 378 (49%) of those travelling to mainland Tanzania, and 17 (59%) of those going outside of the United Republic of Tanzania. Of the travellers to mainland Tanzania with a qPCR result, 232 (61%) were from Magharibi district in Unguja and 255 (67%) were from an index household as opposed to neighbouring/transect households. The majority (80%) were older than 15 years. Members of households in the lower wealth quintiles were underrepresented amongst the travellers (12%) (Table 1 and Additional file 2: Table S1).

**Table 1:** Characteristics of travellers to mainland Tanzania who had a qPCR result

Variables	*n* (%)
Sex	
Male	177 (47)
Female	201 (53)
Age group (years)	
Less than 5	21 (6)
5–15	53 (14)
16–25	107 (26)
26+	197 (52)
Household type	
Index households members	255 (67)
Other households members	123 (33)
District of residence	
Magharibi (Unguja)	232 (61)
Kusini (Unguja)	77 (20)
Micheweni (Pemba)	21 (6)
Chakechake (Pemba)	39 (10)
Mkoani (Pemba)	9 (2)
Occupation	
Enterpreneur	103 (27)
Not employed	133 (35)
Student	102 (27)
Wage Job	40 (11)
Wealth quintile	
Lowest	45 (12)
2nd	65 (17)
Middle	99 (26)
4th	79 (21)
Highest	90 (24)
Highest endemicity category visited	
High	241 (64)
Moderate	45 (12)
Low or very low	92 (24)
Days in high malaria endemicity	
Did not visit high endemicity district	137 (36)
1–7 days	49 (13)
8–14 days	82 (22)
15–30 days	38 (10)
More than 30 days	72 (19)
Season at beginning of trip	
Long rains	40 (11)
Short rains	69 (18)
Dry season	269 (71)
Total	378 (100)

### Prevalence of malaria infection

Among the 601 survey participants with a qPCR result, 21% [95% confidence interval (*CI*) 17–25%] tested positive for malaria. Malaria prevalence was 29% (95% *CI* 23–34%) among travellers to mainland Tanzania, 8% (95% *CI* 4–14%) among those who had travelled within Zanzibar, and 6% (95% *CI* 0.3–61%) among those who had travelled outside of the United Republic of Tanzania. The RDT results (available for 774 travellers) were positive in 6% (95% *CI* 4–8%) of travellers to mainland Tanzania, 4% (95% *CI* 0.6–6%) of those who had travelled within Zanzibar, and 1% (95% *CI* 0.3–5%) in the ones who had travelled outside of the United Republic of Tanzania (Additional file 2: Table S2).

### Travel destinations in mainland Tanzania and travel duration

**Fig. 1:**
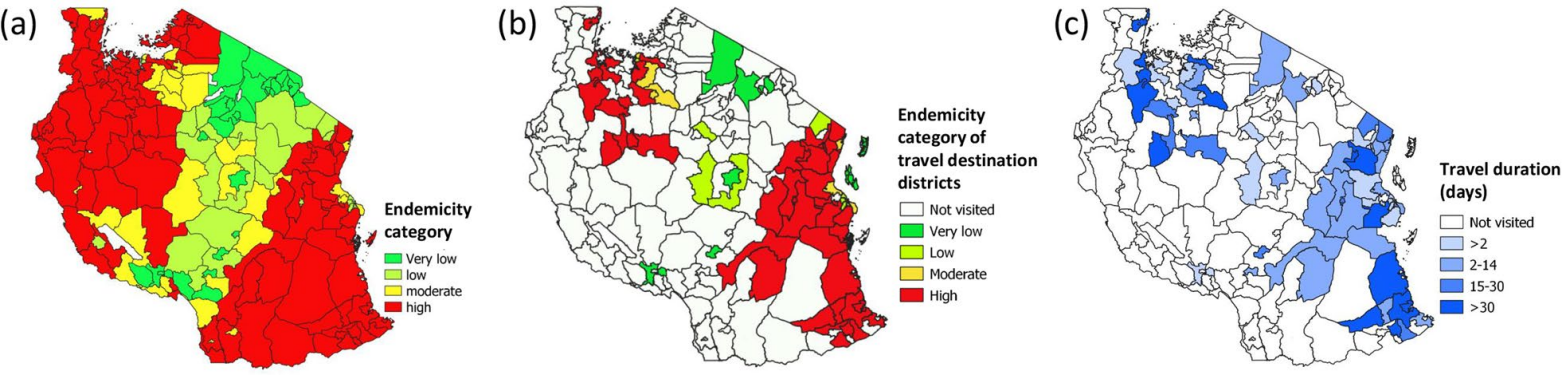
**a** Malaria endemicity categories for 2017/2019; **b** malaria endemicity in districts visited by travellers from Zanzibar; **c** mean travel duration in days

Districts in coastal regions (Dar es Salaam, Tanga and Pwani) of mainland Tanzania were the most commonly visited, followed by districts in Morogoro, in the north-west (Shinyanga, Mwanza, Geita, and Tabora), and in south-east Tanzania (Mtwara and Lindi) (Fig. 1 and Additional file 2: Table S8). A majority (64%) of the 378 travellers to mainland Tanzania reported having visited districts with a high malaria endemicity, 45 (12%) visited districts with moderate endemicity and 92 (24%) went only to districts with low or very low endemicity. A fifth (77/378) of travellers to mainland Tanzania only went to the four low-endemicity councils of Dar es Salaam (Ilala, Kinondoni, Temeke, Ubungo).

The median duration of trips made to mainland Tanzania was 4 days [interquartile range (IQR): 2–4)]. The median number of nights spent in highly endemic districts was 15 days (IQR: 10–58), while the median duration of trips exclusively to low-endemicity areas of Dar es Salaam was 2 days (IQR: 1–9) (Additional file 2: Table S4).

### Travel and malaria infection by qPCR

**Fig. 2:**
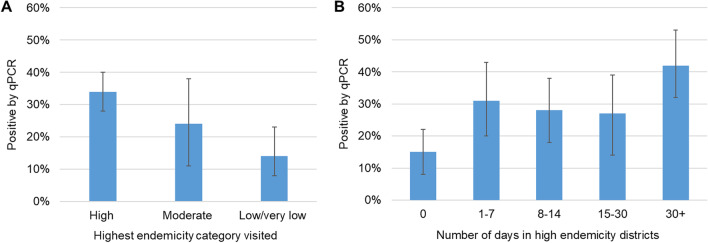
**a** Malaria prevalence in travellers by the highest endemicity category of their trip; **b** Malaria prevalence by the number of days spent in high endemicity districts

The higher the malaria endemicity of the visited districts in mainland Tanzania, the more likely a person was malaria positive (Fig. 2a and Table 2). Travellers who had visited a district with high malaria endemicity contributed 83 (77%) of the 108 infections found in travellers to mainland Tanzania. Individuals who travelled to high endemicity districts for more than thirty days had a higher proportion of positive test results compared to those who spent fewer days in the same areas (Fig. 2a and Additional file 2: Table S7).

**Table 2:** Crude and adjusted odds ratios for factors associated with malaria infection in travellers to mainland Tanzania (*N* = 378)

	qPCR results		Univariable analysis			Multivariable analysis**		
	Negative *n* (%)	Positive *n* (%)	*OR*	95% *CI*	*P*-value	Adjusted *OR*	95% *CI*	*P*-value
Sex								
Males	126 (71)	51 (29)	Ref		0.85	Ref		0.57
Females	144 (72)	57 (28)	1.0	0.6–1.8		0.8	0.4–1.7	
Age groups (years)								
Less than 5	15 (71)	6 (29)	0.9	0.3–2.9	0.36	0.7	0.2–2.8	0.75
5–19	81 (78)	23 (22)	0.6	0.3–1.1		0.5	0.2–1.2	
20–25	38 (68)	18 (32)	1.0	0.5–2.2		0.7	0.3–2.0	
26+	136 (69)	61 (31)	Ref			Ref		
Households types								
Other households members	115 (93)	9 (7)	Ref		< 0.001			
Index households members	156 (61)	99 (39)	14	5.4–41.3				
Districts of residence								
Pemba	51 (74)	18 (26)	0.9	0.4–2.1	0.78	Ref		0.89
Unguja	219 (71)	90 (29)	Ref			1.1	0.3–3.6	
Occupation								
Entrepreneur	60 (58)	43 (42)	Ref		< 0.001			
Not employed	95 (71)	38 (29)	0.5	0.3–1.0				
Wage Job	28 (70)	12 (30)	0.6	0.2–1.4				
Student	87 (85)	15 (15)	0.2	0.1–0.5				
Wealth quintile								
Lowest	32 (71)	13 (29)	1.8	0.5–6.2	0.15	1.2	0.3–5.8	0.28
2nd	47 (72)	18 (28)	1.7	0.6–5.4		1.6	0.4–7.1	
Middle	61 (62)	38 (38)	3.5	1.2–9.6		2.6	0.7–9.7	
4th	60 (76)	19 (24)	1.2	0.4–3.7		0.9	0.2–3.5	
Highest	70 (78)	20 (22)	Ref			Ref		
Highest endemicity district visited								
High	158 (66)	83 (34)	5.4	2.0–14.2	0.002	7.0	1.9–25.5	0.004
Moderate	34 (76)	11 (24)	2.0	0.6–7.1		1.7	0.3–8.5	
Low or very low	78 (85)	14 (15)	Ref			Ref		
*Travel nights by endemicity*								
High endemicity districts								
Did not visit	113 (82)	25 (18)	Ref		0.002			
1–7 days	32 (65)	17 (35)	4.7	1.5–14.6				
8–14 days	62 (76)	20 (24)	1.9	0.7–5.2				
15 –30 days	27 (71)	11 (29)	2.6	0.7–9.1				
More than a month	37 (51)	35 (49)	8.6	3.0–25.1				
High and moderate endemicity districts								
Did not visit	78 (85)	14 (15)	Ref		0.008			
1–7 days	44 (69)	20 (31)	4.2	1.4–13.1				
8–14 days	60 (72)	23 (28)	3.0	1.0–8.8				
15–30 days	36 (73)	13 (27)	2.7	0.8–9.0				
More than a month	52 (58)	38 (42)	8.1	2.6–24.7				
Low endemicity councils of Dar es Salaam region only**								
Did not visit	203 (75)	99 (92)	Ref		0.001			
1–7 days	48 (18)	5 (5)	0.2	0.1–0.6				
8–14 days	6 (2)	1 (1)	0.3	0–2.9				
15–30 days	14 (5)	3 (3)	0.4	0.1–1.6				
*Malaria protective measures during travel*								
Bed net use								
Never used	48 (56)	37 (44)	0.7	0.3–1.6	< 0.001	0.7	0.2–1.8	< 0.001
Used sometimes	13 (52)	12 (48)	1.6	0.4–6.7		1.9	0.4–10.0	
Always used	46 (48)	49 (52)	Ref			Ref		
Missing	163 (94)	10 (6)	0	0–0.1		0	0.0–0.1	
Burn coils								
Never used	95 (52)	87 (48)	1.4	0.3–6.7	< 0.001			
Used sometimes	3 (33)	6 (67)	4.3	0.3–56.2				
Always used	9 (64)	5 (36)	Ref					
Missing	163 (94)	10 (6)	0	0–0.3				
Repellent use								
Never used	89 (51)	86 (49)	1.5	0.4–6.0	< 0.001			
Used sometimes	6 (60)	4 (40)	0.8	0.1–7.7				
Always used	12 (60)	8 (40)	Ref					
Missing	163 (94)	10 (6)	0	0–0.2				
Seasons								
Rainy season	79 (72)	30 (28)	Ref		0.49	Ref		
Dry season	191 (71)	78 (29)	1.3	0.6–2.7		2.7	1.0–7.6	0.05

Classification of endemicity categories according to Thawer et al. [33]

*Multivariable logistic regression model with household as random effect

**Excluding Kigamboni district council (moderate endemicity category)

A subset of 205 travelers to mainland Tanzania had data on the use of protective measures during their trip. Of those, 121 (58%) reported using bed nets, 23 (11%) burnt mosquito coils and 30 (15%) used topical repellents at some stage during their travels (Additional file 2: Table S5). There was no evidence of a difference in net use by season (*P* = 0.24) or by endemicity category of the destination districts (*P* = 0.25) (Additional file 2: Table S6).

Relative to individuals travelling to districts with lower endemicity, travelling to districts with a high malaria endemicity was associated with a seven-fold increase in the odds of malaria infection [adjusted odds ratio (*OR*) = 7.0, 95% *CI* 1.9–25.5, *P* = 0.003] (Table 2) adjusting for the participant’s background characteristics, season and net use. Those travelling in the dry season had almost three times higher odds of being PCR positive than those travelling in the rainy season (adjusted *OR* = 2.7, 95% *CI* 0.98–7.55, *P* = 0.054). Other covariates were not significantly associated with malaria infection (Table 2).

## Discussion

Addressing malaria importation in a near-elimination setting requires an understanding of how and from where parasites are imported. Our study provides details on travel patterns between the low transmission setting of Zanzibar and mainland Tanzania. It identifies an increased risk of malaria infection in those Zanzibar residents who travel to districts in mainland Tanzania that are classified as highly malaria endemic based on a national malaria risk stratification [33]. These findings can inform the development of tailored approaches to target the importation of malaria parasites into Zanzibar using data that can be collected routinely.

While residual local transmission persists in Zanzibar, with *Anopheles arabiensis* being the most abundant vector [36], previous analyses of malaria data have consistently identified travel to mainland Tanzania as a risk factor for malaria infection among residents of Zanzibar [18, 23, 26, 30, 37]. Our results complement these studies by providing details about the precise travel destination and the likely origin of the malaria infections in Tanzania mainland [23, 38]. Genotypic analyses of our data published elsewhere [39] provide evidence of local transmission between people without reported travel history, and of local onward transmission that originated from reportedly imported infections and persisted over a period of several months. The dominant local vector, *An. arabiensis*, was found to bite preferentially outdoors [36], making it less susceptible to current indoor vector control interventions such as indoor residual spraying and insecticide-treated nets, potentially driving persistent malaria transmission in Zanzibar. While the probability of transmission from individuals with low-density infections is generally lower [40, 41], subpatent infections have been found to later result in a higher density, potentially symptomatic infection, increasing the probability of transmission [42–44]. Furthermore, low-density chronic infections may be associated with other sequelae, such as anaemia [45]. In light of the persisting receptivity of Zanzibar and ongoing local transmission [23, 30, 36], identifying and eliminating both patent and subpatent infections can therefore contribute to preventing morbidity and onward transmission, both of which should be considered essential in the frame of a local malaria elimination programme.

Malaria endemicity remains high in the south-east of Tanzania, including the Indian Ocean coastline south of Dar es Salaam, and in the north-west of the country [33]. Roughly two-thirds of the trips reported by our study participants from Zanzibar included visiting a district classified as highly malaria endemic according to Thawer et al. [33] while few travelers exclusively visited areas with a low malaria endemicity (which includes the centre and north of Tanzania, as well as most of Dar es Salaam). Travelers who visited highly endemic districts contributed the majority (77%) of all infections detected among travellers to mainland Tanzania. Trips to high endemicity areas were not only more frequent but also generally longer than those to other areas (including Dar es Salaam, the main port of entry to mainland Tanzania), which was expected to result in an increased chance of exposure to infective mosquito bites.

Previous studies applied mobile phone data to capture the movement of travellers between Zanzibar and mainland Tanzania and model the risk of importing parasites based on travel destinations in five large telecommunication coverage regions of the mobile phone service provider ‘Zantel’ in mainland Tanzania [24, 25]. Yet, these coverage regions are very large and comprise a variety of endemicity settings. For example, the Dar es Salaam coverage region, previously identified as the primary travel destination from Zanzibar [24], comprised the entire coastline from the Kenyan to the Mozambican border. While Dar es Salaam is where most of the travellers from Zanzibar start their journey to other destinations in mainland Tanzania, our study established that only 20% of travellers remain in Dar es Salaam without travelling onwards to other destinations, and of those who did stay, only a few were infected with malaria parasites.

Our study combined self-reported travel data that can be collected routinely in RACD household follow-up activities by ZAMEP and malaria endemicity data based on routine health information system data from mainland Tanzania [46]. The advantages of our approach include the higher granularity of the travel data, reflecting the heterogeneity of malaria endemicity in mainland Tanzania, and the continuous data availability through routinely implemented surveillance activities. The strong association between malaria endemicity in the travel destinations and infection among travellers observed in our analysis, provides a clear rationale for prioritising those who travel to highly endemic areas with more strict measures to prevent malaria importation in Zanzibar. At the same time, it underlines the importance of reducing malaria transmission in high endemicity areas in mainland Tanzania in order to reduce parasite importation in the isles, as also suggested in a recent modelling study [26].

Considering that Dar es Salaam is the main port of entry and exit for travellers between Zanzibar and mainland Tanzania and an important commercial centre connecting regions within Tanzania and neighbouring countries, measures to reduce transmission must include preventing the invasion of more aggressive urban malaria vector species, namely *An. stephensi*, that could also be carried to Zanzibar via different modes of human or cargo transport [47]. In light of the high connectivity of Zanzibar and mainland Tanzania, both vector and parasite surveillance and adequate targeted response should be a priority for both the mainland Tanzania national malaria control programme and ZAMEP, working closely together.

Albeit available only for a sub-set of travellers recruited in the later part of the study, our data indicates that travellers were not likely to consistently use mosquito nets or other measures to prevent mosquito bites while travelling. Although there is a high mosquito net coverage in mainland Tanzania (84% use in 9–19 year old pupils) [48], households may not have sufficient (spare) nets for temporary guests. Travellers would have to carry their own protective gear if it is not provided where they stay overnight. Risk awareness or personal preferences may further influence the use of protective measures even if they are available [49]. Our findings suggest that malaria infection and importation in Zanzibar have an association with travelling during the dry season. In Zanzibar, there is a strong relationship between seasonality and malaria transmission peaks, as a significant proportion of infections occur towards the end of the rainy seasons. The slightly lower use of mosquito nets during the dry seasons, when the temperatures are likely to be higher outside and in the room and sleeping space, may be due to discomfort when sleeping under the net, as previously reported elsewhere [50]. The observed increase in the odds of infection during the rainy season can be attributed to the timing of data collection. This is due to the fact that there were only a few observations in the rainy seasons.

The analyses in this manuscript used self-reported travel information over a period of 60 days with broad categories of number of days travelled in the analysis of travel duration. Data collection for this study was linked to ZAMEP’s reactive case detection, that is, each data collection activity was triggered by a reported confirmed malaria case. Even though the sample included transect households (with a malaria prevalence comparable to the general population [23]), households with travellers who returned without a malaria infection might be underrepresented in this study, potentially biasing the sample towards travel to higher endemicity areas and higher malaria prevalence than that found in the general population. While the frequency of travel destinations might therefore not be representative of all travellers from Zanzibar, the destinations are likely to be an adequate reflection of locations from which malaria infections are imported. At the same time, our study only included five districts of Zanzibar [excluding, for example, Mjini (Urban), the district with the highest number of travellers according to routine ZAMEP data, and possibly the highest number of imported infections [25]]. Hence some of the results may not be representative of all of Zanzibar. Travel destination data collected by ZAMEP through the routine surveillance-response system may be used to update our analyses of travel destinations, classified based on their level of endemicity. The use of qPCR for diagnosing infections in our study, rather than RDT, as in routine case management and surveillance, results in a higher sensitivity and specificity as qPCR detects lower density infections [31] and does not provide false-positive results due to the prolonged HRP2 antigenaemia [51]. Analyses based on routinely collected data may therefore differ slightly from our study results. A qPCR result was not available from all surveyed individuals, partly due to their non-availability during sampling, and due to a density cut-off introduced during screening, as described in Grossenbacher et al. [31]. Neither of these reasons for non-availability of a qPCR result are likely to be linked to the probability of being infected among travelers. Even though there was a slightly larger proportion of samples available from the second study year (273/528 in 2018 vs 105/241 in 2017) and from Magharibi compared to the other study districts (232/527 vs 146/242), it is unlikely that the magnitude of the difference would compromise the generalizability of our findings.

Based on our findings, interventions addressing malaria importation could preferentially target individuals who travel to and from high endemicity areas in mainland Tanzania. Regular analyses of surveillance data indicating the origin of infections classified as ‘imported’ and further qualitative research may be needed to identify socio-demographic characteristics of these ‘high risk’ groups and to develop and implement effective targeted interventions. Similarly, additional in-depth investigations of drivers of local transmission could help to identify high risk groups who are susceptible to contracting infections locally in Zanzibar. Interventions to prevent and clear imported malaria infections, for example malaria chemoprophylaxis, sensitization activities, or test-and-treat programmes at the port of entry, may be more feasible and cost-effective to implement in clearly defined ‘high risk’ groups rather than all incoming travellers.

## Conclusions

As Zanzibar is aiming to eliminate malaria, the key challenge of malaria parasite importation needs to be addressed. Our study identified specific travel patterns that increased the risk of malaria infection. Measures to reduce importation would benefit from identified ‘high risk’ population groups for targeted interventions. By identifying population groups with similar characteristics and travel patterns, targeted interventions may be more effective in preventing and clearing infections during and after travel than a blanket approach targeting all travellers.

### Supplementary Information

**Additional file 1: Figure S1.** Study flow chart and description of the study population.

**Additional file 2: Table S1.** Study population by travel destination and socio-demographic characteristics. **Table S2.** Prevalence of malaria infection. **Table S3.** Number of travellers to mainland Tanzania by highest endemicity category of visited districts. **Table S4.** Median and IQR of the number of nights spent, by endemicity category (N = 378).** Table S5.** Malaria protective measures during travel N = 205. **Table S6.** Malaria protective measures during travel N = 205. **Table S7.** Adjusted odds ratios for factors associated with malaria infection in travellers to mainland Tanzania (N = 378). **Table S8**. Prevalence of malaria infection by districts visited.

## Data Availability

All the relevant datasets relating to this study are available upon reasonable request to the co-authors.

## Abbreviations

ACT: Artemisinin-based combination therapy
*CI*: Confidence interval
DMSO: District Malaria Surveillance Officer
*OR*: Odds ratio
qPCR: Quantitative polymerase chain reaction
RACD: Reactive case detection
Ref: Reference category
RDT: Rapid diagnostic test
ZAMEP: Zanzibar Malaria Elimination Programme
