# Supplementary material for: Risk of imported malaria infections in Zanzibar: a cross-sectional study
Source: Infect Dis Poverty. 2023 Aug 28;12:80. doi: 10.1186/s40249-023-01129-5 (PMC10464242; doi:10.1186/s40249-023-01129-5)
Supplement: Supplementary file 1 — Additional file 1: Figure S1. Study flow chart and description of the study population. [file 40249_2023_1129_MOESM1_ESM.docx]

**Supplementary figure**

**Risk of imported malaria infections in Zanzibar: a cross-sectional study**

Bakar S. Fakih^1,2,3*^; Aurel Holzschuh^2,4^; Amanda Ross^2,3^; Logan Stuck^5,6^; Ramadhan Abdul^1,6^; Abdul-Wahid H. Al-Mafazy^7,^; Imani Irema^1^; Abdallah Mbena^1^; Sumaiyya G. Thawer^2,3^; Shija J. Shija^7^; Safia M. Aliy^7^; Abdullah Ali^7^; Günther Fink^2,3^; Joshua Yukich^5^; Manuel W. Hetzel^2,3^

^1^ Ifakara Health Institute, Dar es Salaam, Tanzania

^2^ Swiss Tropical and Public Health Institute, Allschwil, Switzerland

^3^ University of Basel, Basel, Switzerland

^4^ Department of Biological Sciences, Eck Institute for Global Health, University of Notre Dame, United States

^5^ Tulane University School of Public Health and Tropical Medicine, New Orleans, Louisiana, United States

^6^ Current affiliation: Amsterdam Institute for Global Health and Development, Amsterdam, Netherlands

^7^ Zanzibar Malaria Elimination Programme, Zanzibar, United Republic of Tanzania

*Corresponding author: Bakar Shariff Fakih: bakar.fakih@swisstph.ch, bfakih@ihi.or.tz


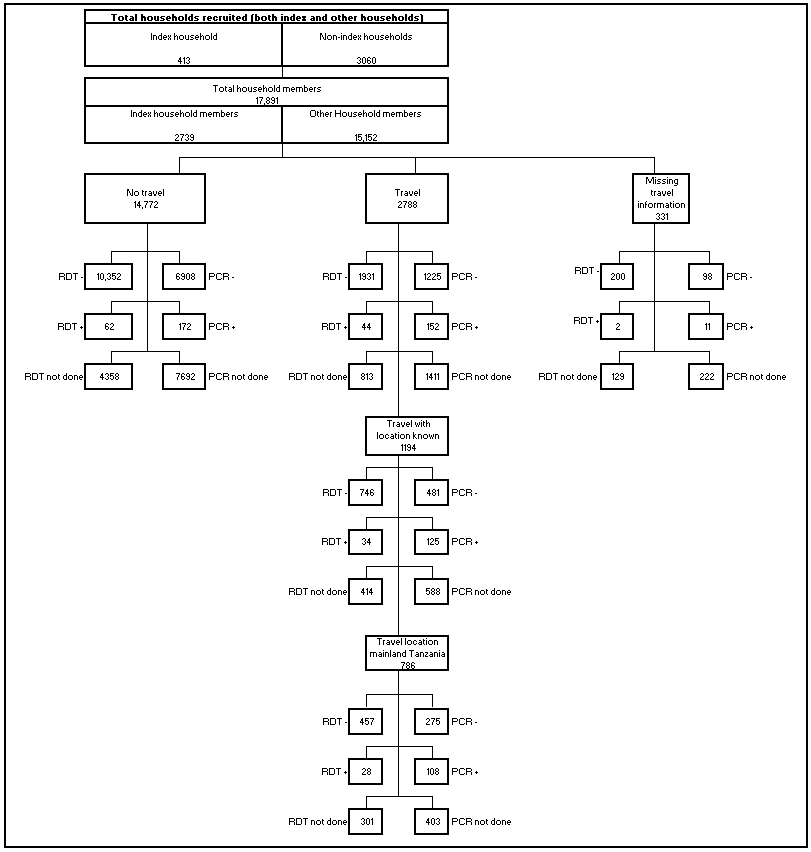


***Figure S1:*** *Study flow chart and description of the study population*
