# Supplementary material for: Risk of imported malaria infections in Zanzibar: a cross-sectional study
Source: Infect Dis Poverty. 2023 Aug 28;12:80. doi: 10.1186/s40249-023-01129-5 (PMC10464242; doi:10.1186/s40249-023-01129-5)
Supplement: Supplementary file 2 — Additional file 2: Table S1. Study population by travel destination and socio-demographic characteristics. Table S2. Prevalence of malaria infection. Table S3. Number of travellers to mainland Tanzania by highest endemicity category of visited districts. Table S4. Median and IQR of the number of nights spent, by endemicity category (N = 378). Table S5. Malaria protective measures during travel N = 205. Table S6. Malaria protective measures during travel N = 205. Table S7. Adjusted odds ratios for factors associated with malaria infection in travellers to mainland Tanzania (N = 378). Table S8. Prevalence of malaria infection by districts visited. [file 40249_2023_1129_MOESM2_ESM.docx]

**Supplementary tables**

**Risk of imported malaria infections in Zanzibar: a cross-sectional study**

Bakar S. Fakih^1,2,3*^; Aurel Holzschuh^2,4^; Amanda Ross^2,3^; Logan Stuck^5,6^; Ramadhan Abdul^1,6^; Abdul-Wahid H. Al-Mafazy^7,^; Imani Irema^1^; Abdallah Mbena^1^; Sumaiyya G. Thawer^2,3^; Shija J. Shija^7^; Safia M. Aliy^7^; Abdullah Ali^7^; Günther Fink^2,3^; Joshua Yukich^5^; Manuel W. Hetzel^2,3^

^1^ Ifakara Health Institute, Dar es Salaam, Tanzania

^2^ Swiss Tropical and Public Health Institute, Allschwil, Switzerland

^3^ University of Basel, Basel, Switzerland

^4^ Department of Biological Sciences, Eck Institute for Global Health, University of Notre Dame, United States

^5^ Tulane University School of Public Health and Tropical Medicine, New Orleans, Louisiana, United States

^6^ Current affiliation: Amsterdam Institute for Global Health and Development, Amsterdam, Netherlands

^7^ Zanzibar Malaria Elimination Programme, Zanzibar, United Republic of Tanzania

*Corresponding author: Bakar Shariff Fakih: bakar.fakih@swisstph.ch, bfakih@ihi.or.tz

***Table S1:*** *Study population by travel destination and socio-demographic characteristics*

| **Socio-demographic characteristics** | Travel within Zanzibar  n (%) | Travel to mainland Tanzania  n (%) | Travel outside Tanzania n (%) |
| --- | --- | --- | --- |
| **Sex** |  |  |  |
| Male | 81 (39) | 177 (47) | 7 (41) |
| Female | 125 (61) | 201 (53) | 10 (59) |
|  |  |  |  |
| **Age group (years)** |  |  |  |
| Less than 5 | 15 (7) | 21 (6) | – |
| 5–15 | 36 (17) | 53 (14) | 3 (18) |
| 16–25 | 47 (23) | 107 (28) | 5 (29) |
| 26 + | 108 (52) | 197 (52) | 9 (53) |
|  |  |  |  |
| **Household type** |  |  |  |
| Index household members | 35 (17) | 255 (67) | 15 (88) |
| Other household members | 171 (83) | 123 (33) | 2 (12) |
|  |  |  |  |
| **District of residence** |  |  |  |
| Magharibi (Unguja) | 47 (23) | 232 (61) | – |
| Kusini (Unguja) | 63 (31) | 77 (20) | 1 (6) |
| Micheweni (Pemba) | 31 (15) | 21 (6) | 16 (94) |
| Chakechake (Pemba) | 52 (25) | 39 (10) | – |
| Mkoani (Pemba) | 13 (6) | 9 (3) | – |
|  |  |  |  |
| **Occupation** |  |  |  |
| Entrepreneur | 61 (30) | 103 (27) | 5 (29) |
| Wage Job | 7 (3) | 40 (11) | 1 (6) |
| Student | 51 (25) | 102 (27) | 6 (35) |
| Not employed | 87 (42) | 133 (35) | 5 (29) |
|  |  |  |  |
| **Wealth quintile of household** |  |  |  |
| Lowest | 30 (15) | 45 (12) | 6 (35) |
| 2nd | 45 (23) | 65 (17) | – |
| Middle | 57 (28) | 99 (26) | – |
| 4th | 41 (20) | 79 (21) | 11 (65) |
| Highest | 33 (16) | 90 (24) | – |
| **Total** | **206 (100)** | **378 (100)** | **17 (100)** |

***Note:*** *Includes only study participants with available qPCR data*

***Table S2:*** *Prevalence of malaria infection*

|  | **RDT results** | | |  | **qPCR results** | | |
| --- | --- | --- | --- | --- | --- | --- | --- |
|  | N | N positive | % (95% *CI*) |  | N | N positive | % (95% *CI*) |
| **General location** |  |  |  |  |  |  |  |
| Zanzibar | 275 | 5 | 2 (0.6–6) |  | 206 | 16 | 8 (4–14) |
| Mainland | 479 | 28 | 6 (4–8) |  | 378 | 108 | 29 (23–34) |
| Outside Tanzania | 20 | 1 | 1 (0.3–5) |  | 17 | 1 | 6 (0.3–61) |

***Table S3:*** *Number of travellers to mainland Tanzania by highest endemicity category of visited districts*

| **Endemicity category** | **N (%)** |
| --- | --- |
| High | 241 (64) |
| Moderate | 45 (12) |
| Low to very low | 92 (24) |

***Table S4:*** *Median and IQR of the number of nights spent, by endemicity category (****N=378*)**

| **Endemicity category of travel destination** | median | IQR |
| --- | --- | --- |
| Mainland | 4 | 2–4 |
| High | 15 | 10–58 |
| Moderate–high | 14 | 7–44 |
| Low to very low | 3 | 1–14 |
| Dar es Salaam only* | 2 | 1–9 |
| Zanzibar | 7 | 3–16 |
| Outside of the United Republic of Tanzania | 14 | 8–34 |

* Four councils excluding Kigamboni

***Table S5:*** *Malaria protective measures during travel N=205*

| **Bednet use** | n (%) |
| --- | --- |
| Never | 85 (41) |
| Always/sometimes | 120 (59) |
| **Burn coils** |  |
| Never | 182 (89) |
| Always/sometimes | 23 (11) |
| **Repellent use** |  |
| Never | 175 (85) |
| Always/sometimes | 30 (15) |

***Table S6:*** *Malaria protective measures during travel N=205*

| **Bed net use in different endemicity categories** | High | Moderate | Low and very low |
| --- | --- | --- | --- |
| Never | 54 (42) | 7 (29) | 24 (47) |
| Sometimes | 17 (13) | 1 (4) | 7 (14) |
| Always | 59 (45) | 16 (67) | 20 (39) |
|  |  |  |  |

***Table S7:*** *Adjusted odds ratios for factors associated with malaria infection in travellers to mainland Tanzania (N = 378)*

|  | **Adjusted OR** |  | **95% *CI*** | ***P*-value** |
| --- | --- | --- | --- | --- |
| **Number of nights in high endemicity districts** | | | | |
| Did not visit | Ref |  |  | 0.02 |
| Slept 1–7 days | 6.9 |  | 1.5–32.5 | |
| Slept 8–14 days | 3.5 |  | 0.9–13.9 | |
| Slept 15–30 days | 4.9 |  | 0.9–27.6 | |
| More than a month | 9.5 |  | 2.1–42.6 | |
| **Travellers slept under net** | |  |  |  |
| Always used net | Ref |  |  | <0.001 |
| Never used net | 0.7 |  | 0.2–1.9 |  |
| Sometimes used net | 1.8 |  | 0.4–9.5 |  |
| Missing | 0 |  | 0–0.1 |  |
| **Age group** |  |  |  |  |
| Less than 5 years | Ref |  |  | 0.74 |
| 5–19 | 0.7 |  | 0.2–3.6 |  |
| 20–25 | 1.1 |  | 0.2–5.6 |  |
| Above 25 | 1.6 |  | 0.4–6.7 |  |
| **Sex** |  |  |  |  |
| Males | Ref |  |  |  |
| Females | 0.8 |  | 0.4–1.7 |  |
| **Wealth quintile** |  |  |  |  |
| Lowest | 1.3 |  | 0.3–6.3 |  |
| 2nd | 1.7 |  | 0.4–7.9 |  |
| Middle | 2.6 |  | 0.7–9.8 |  |
| 4th | 1.0 |  | 0.2–4.0 |  |
| Highest | Ref |  |  | 0.39 |
| **Island of residence** |  |  |  |  |
| Pemba | Ref |  |  |  |
| Unguja | 1.0 |  | 0.3–3.5 |  |
| **Seasons** |  |  |  |  |
| Rainy season | Ref |  |  |  |
| Dry season | 2.6 |  | 0.9–7.3 |  |

***Table S8:*** *Prevalence of malaria infection* *by districts visited*

| **Region** | **District** | **Endemicity category** | **N tested** | **N positive (%)** |
| --- | --- | --- | --- | --- |
| Arusha | Arusha City Council | Very low | 3 | 3 (100) |
| Arusha | Meru District Council | Very low | 1 | 0 |
| Dar es Salaam | Ilala Municipal Council | low | 21 | 0 |
| Dar es Salaam | Kigamboni Municipal Council | moderate | 18 | 4 (22) |
| Dar es Salaam | Kinondoni Municipal Council | low | 18 | 2 (11) |
| Dar es Salaam | Temeke Municipal Council | low | 41 | 7 (17) |
| Dar es Salaam | Ubungo Municipal Council | low | 78 | 0 |
| Dodoma | Dodoma Municipal Council | Very low | 1 | 0 |
| Geita | Bukombe District Council | high | 10 | 2 (20) |
| Geita | Chato District Council | high | 3 | 2 (67) |
| Iringa | Mafinga Town Council | Very low | 1 | 0 |
| Lindi | Kilwa District Council | high | 11 | 3 (27) |
| Lindi | Lindi D Council | high | 2 | 1 (50) |
| Lindi | Lindi Municipal Council | high | 7 | 6 (86) |
| Lindi | Nachingwea District Council | high | 1 | 0 |
| Lindi | Ruangwa District Council | high | 3 | 2 (67) |
| Morogoro | Kilombero District Council | high | 11 | 2 (18) |
| Morogoro | Kilosa District Council | high | 4 | 2 (50) |
| Morogoro | Morogoro District Council | high | 13 | 2 (15) |
| Morogoro | Morogoro Municipal Council | low | 6 | 5 (83) |
| Morogoro | Mvomero District Council | high | 8 | 1 (13) |
| Morogoro | Ulanga District Council | high | 8 | 2 (25) |
| Mtwara | Masasi District Council | high | 1 | 1 (100) |
| Mtwara | Newala Town Council | high | 3 | 1 (33) |
| Mtwara | Tandahimba District Council | high | 9 | 3 (33) |
| Mwanza | Ilemela Municipal Council | moderate | 3 | 3 (100) |
| Mwanza | Magu District Council | high | 3 | 3 (100) |
| Mwanza | Misungwi District Council | high | 2 | 1 (50) |
| Mwanza | Nyamagana Municipal Council | low | 7 | 2 (29) |
| Njombe | Makambako Town Council | Very low | 1 | 1 (100) |
| Pwani | Bagamoyo District Council | moderate | 8 | 4 (50) |
| Pwani | Kibaha District Council | high | 1 | (100) |
| Pwani | Mkuranga District Council | high | 19 | 6 (32) |
| Pwani | Rufiji District Council | high | 13 | 8 (62) |
| Shinyanga | Kahama Town Council | high | 1 | 0 |
| Shinyanga | Kishapu District Council | moderate | 1 | 1 (100) |
| Shinyanga | Shinyanga District Council | high | 1 | 0 |
| Shinyanga | Shinyanga Municipal Council | moderate | 8 | 1 (13) |
| Singida | Manyoni District Council | low | 5 | 1 (20) |
| Tabora | Nzega District Council | high | 8 | 3 (38) |
| Tabora | Urambo District Council | high | 2 | 1 (50) |
| Tabora | Uyui District Council | high | 15 | 5 (33) |
| Tanga | Korogwe District Council | high | 2 | 1 (50) |
| Tanga | Lushoto District Council | low | 2 | 1 (50) |
| Tanga | Mkinga District Council | high | 11 | 2 (18) |
| Tanga | Muheza District Council | high | 19 | 5 (26) |
| Tanga | Pangani District Council | high | 27 | 5 (19) |
| Tanga | Tanga City Council | moderate | 36 | 11 (31) |
